# Palmitoylation is required for TNF-R1 signaling

**DOI:** 10.1186/s12964-019-0405-8

**Published:** 2019-08-05

**Authors:** Philipp Zingler, Vinzenz Särchen, Timo Glatter, Lotta Caning, Carina Saggau, Rahul S. Kathayat, Bryan C. Dickinson, Dieter Adam, Wulf Schneider-Brachert, Stefan Schütze, Jürgen Fritsch

**Affiliations:** 1 Institute of Immunology, Christian-Albrechts-University of Kiel, Kiel, Germany; 2 Facility for Mass Spectrometry and Proteomics, MPI for Terrestrial Microbiology, Marburg, Germany; 3 Department of Chemistry, University of Chicago, Chicago, USA; 4 Department of Infection Prevention and Infectious Diseases, University of Regensburg, Franz-Josef-Strauss Allee 11, 93053 Regensburg, Germany

**Keywords:** Palmitoylation, TNF-R1, Death receptor, Signal transduction, NFκB, Compartmentalization

## Abstract

**Background:**

Binding of tumor necrosis factor (TNF) to TNF-receptor 1 (TNF-R1) can induce either cell survival or cell death. The selection between these diametrically opposed effects depends on the subcellular location of TNF-R1: plasma membrane retention leads to survival, while endocytosis leads to cell death. How the respective TNF-R1 associated signaling complexes are recruited to the distinct subcellular location is not known. Here, we identify palmitoylation of TNF-R1 as a molecular mechanism to achieve signal diversification.

**Methods:**

Human monocytic U937 cells were analyzed. Palmitoylated proteins were enriched by acyl resin assisted capture (AcylRAC) and analyzed by western blot and mass spectrometry. Palmitoylation of TNF-R1 was validated by metabolic labeling. TNF induced depalmitoylation and involvement of APT2 was analyzed by enzyme activity assays, pharmacological inhibition and shRNA mediated knock-down. TNF-R1 palmitoylation site analysis was done by mutated TNF-R1 expression in TNF-R1 knock-out cells. Apoptosis (nuclear DNA fragmentation, caspase 3 assays), NF-κB activation and TNF-R1 internalization were used as biological readouts.

**Results:**

We identify dynamic S-palmitoylation as a new mechanism that controls selective TNF signaling. TNF-R1 itself is constitutively palmitoylated and depalmitoylated upon ligand binding. We identified the palmitoyl thioesterase APT2 to be involved in TNF-R1 depalmitoylation and TNF induced NF-κB activation. Mutation of the putative palmitoylation site C248 interferes with TNF-R1 localization to the plasma membrane and thus, proper signal transduction.

**Conclusions:**

Our results introduce palmitoylation as a new layer of dynamic regulation of TNF-R1 induced signal transduction at a very early step of the TNF induced signaling cascade. Understanding the underlying mechanism may allow novel therapeutic options for disease treatment in future.

**Electronic supplementary material:**

The online version of this article (10.1186/s12964-019-0405-8) contains supplementary material, which is available to authorized users.

## Background

Tumor necrosis factor alpha (TNF) regulates a variety of cellular processes ranging from inflammation, proliferation, to differentiation, and can induce various forms of cell death. TNF signal transduction occurs via binding of the ligand to two different receptors: TNF-R1 and TNF-R2, two members of the TNF-receptor superfamily. TNF-R1 belongs to the subgroup of death receptors (DR) and can induce cell death via its C-terminal “death domain” (DD). The selective recruitment of distinct adaptor proteins to the activated TNF-R1 determines whether cell survival or cell death signaling prevails [1]. Immediately upon ligand binding, the “complex I” adaptor proteins TRADD, RIP1, TRAF2, and c-IAP1 are recruited to the DD of TNF-R1. Signaling from “complex I” is regulated by ubiquitination and finally triggers NF-κB nuclear translocation [1–3]. We and others have shown that this initial signaling cascade is based on plasma membrane resident TNF-R1. One mechanism that shifts the system toward cell death signaling is K63-ubiquitination of TNF-R1, leading to its internalization by formation of TNF-containing endosomes (receptosomes) [4–8]. TNF-R1 internalization triggers K48-ubiquitination and subsequent proteasomal degradation of TRAF2 and RIP1, followed by “complex II” formation by the recruitment of the “death inducing signaling complex” (DISC) proteins FADD and caspase-8 [4, 5, 9, 10]. TNF-receptosomes are also a source for the production of reactive oxygen species by recruitment of riboflavin kinase [11]. Together, these events are the initial triggers for cell death.

Intracellular trafficking of TNF-receptosomes and fusion with trans-Golgi vesicles facilitates maturation towards a multivesicular/lysosomal compartment. Here, a proteolytic cascade leads to the formation of ceramide via acid sphingomyelinase (aSMase) and translocation of active cathepsin D (CtsD) into the cytoplasm. CtsD cleaves and then degrades the anti-apoptotic chaperone HSP90, and activates BID by truncation to tBID. tBID is involved in mitochondrial outer membrane permeabilization and cell death [12–14]. Many players involved in the regulation of TNF-R1 signal transduction are known (for review, see [1, 15, 16]). What remains enigmatic is: How are these proteins recruited to a distinct subcellular localization (i.e. discrete membrane domains or compartments) to form functional protein complexes at the right time upon TNF stimulation?

The reversible post-translational protein modification of cysteine residues with a palmitic acid through thioester formation (S-palmitoylation) is known to modulate target protein interactions with lipids and with other proteins. The palmitoyl group is attached to a target protein by palmitoyl acyltransferases (PAT) containing the characteristic Asp-His-His-Cys (DHHC) motif. In mammals, 23 zDHHC containing proteins have been identified. Palmitoylation is fully reversible and depalmitoylation is catalyzed by palmitoyl thioesterases (PTE). The best-described PTEs are the cytosolic APT1, APT2, and the lysosomal PPT1, but the family is growing, e.g. through the identification of the ABDH17 proteins [17–19]. Palmitoylation of proteins can have various different functions and has an impact on different biological processes and diseases, as summarized in various extensive reviews [20, 21].

Here, we identify palmitoylation as a novel molecular switch that modulates TNF-R1 mediated signaling. Our initial observations that pharmacological interference with palmitoylation modulates TNF-R1 internalization prompted us to investigate how lipidation regulates TNF-R1 signaling. We focused on the palmitoylation state of TNF-R1, showing that its de-palmitoylation in response to TNF is mediated by the PTE APT2. Knock down of APT2 enhances apoptosis but blocks signaling via NF-κB. Mutagenesis of the putative palmitoylation site C248 altered TNF-R1 surface expression resulting in both, reduced cell death and NF-κB signaling.

## Methods

PIC: cOmplete protease inhibitor cocktail (Roche).

### Antibodies

Cell Signaling: anti-CD71 (#13113), anti-cleaved Caspase 3 (#9661S), anti-His (#2365), anti-IκBα (#4814), anti-Integrinα6 (#3750), anti-STX6 (#2869), anti-TNF-R1 (#3736), anti-PARP (#9542S).

ENZO: anti-Ceramide (15B4; ALX-804-196-T050).

LSbio: anti-Lypla2/APT2 (LS-C158086).

LifeTechnologies: anti-mouse Alexafluor488 (A21202).

Millipore: anti-mouse light chain (AP200P), anti-rabbit light chain (MAB201P).

Proteintech: anti-βActin (HRP-60008), anti-GAPDH (HRP-60004).

Santa Cruz Biotechnology: anti-Rab5B (sc-598).

Sigma-Aldrich: anti-PPT1 (HPA021546).

ThermoFisher: anti-Lypla1/APT1 (PA5–28034).

### Cell culture

Human U937 cells (DSMZ Braunschweig, Germany) were maintained in RPMI 1640 medium (Gibco, Life Technologies) supplemented with 5% v/v FCS (Gibco, Life Technologies) and 1% v/v Pen/Strep (Merck Millipore) under standard cell culture conditions.

### TNF-R1 knock-out by CRISPR/Cas9

CRISPR/Cas9 plasmids (Sigma-Aldrich Target ID1: HS0000079005; Target ID2: HS0000079009) were nucleofected (Lonza). After transfection cells were FACS sorted for GFP positive signal and single clones were isolated and further characterized.

### Generation of TNF-R1 constructs and generation of virus particles

FLAG-TNF-R1_C248S_ was ordered from Geneart and cloned into pMOWS vector (validated by sequencing: pMOWSfwd 5′-TATCCAGCCCTCACTCCTTCTCTAG-3′; pMOWSrev 5′-CCACATAGCGTAAAAGGAGCAAC-3′). To generate virus particles, vector was transfected to Gryphon™ cells (Allele Biotechnology) using lipofectamine 2000. After two days, virus containing supernatant was centrifuged (450 x g, 4 °C, 5 min) sterile filtrated (0.45 μm) and added to 0.5 × 10^6^ ΔTNF-R1 U937 cells in the presence of 5 μg/ml Polybrene. Next day, medium changed to standard cell culture medium and 1.5 μg/ml puromycinwas added after 24 h.

### Internalization assay

TNF-R1 internalization was analyzed by imaging flow cytometry (ISX MK2, Amnis/EMD Millipore). For this 10^6^ cells/sample were incubated with biotinylated TNF (NFTA0, Biotechne) coupled to streptavidin Alexafluor488 (Life Technologies) on ice for 20 min, followed by synchronized receptor internalization at 37 °C. Where inhibitors were used, these were pre-incubated for 20 min at room temperature followed by the incubation on ice. Internalization was stopped by addition of cold PBS / cell mask (dilution 1:20,000; Life Technologies), incubation for 5 min on ice sedimentation and fixation of the cells in 2% PFA/PBS. At least 5000 images per experiment were acquired using the Inspire software (200.1.388.0) and analyzed using the Amnis IDEAS software (6.0.154.0).

### Analysis of protein surface expression

Cells were labeled as for internalization measurement. At least 5000 images per experiment were acquired using the Inspire software (200.1.388.0) and fluorescence intensity of Ch2 (Alexafluor488) was analyzed using Amnis IDEAS software (6.0.154.0).

### Apoptosis assay

For apoptosis measurement by imaging flow cytometry, cells were incubated for the times indicated in the figure with TNF (100 ng/ml) under standard cell culture conditions. 30 min before end Hoechst stain (Sigma-Aldrich) was added to the culture medium finally diluted 1:10,000. Up to 10,000 images were captured per assay and quantified using Amnis IDEAS software (6.0.154.0) .

### PTE activity assay

PTE activity was analyzed by imaging flow cytometry (Amnis/EMD Millipore). In brief, 10^6^ U937 cells were pre-incubated for 10 min at room temperature followed by 10 min on ice with DPP-2 or DPP-3 fluorescent probes (provided by B.C. Dickinson) [22]. The APT2 selective inhibitor ML349 (#5344, bio-techne), the APT1 selective inhibitor ML349 (#5345, bio-techne) and the pan PTE inhibitor Palmostatin B (Sigma-Aldrich) were used as controls. TNF was added and incubated for another 20 min on ice. Activation was triggered by temperature shift to 37 °C for the indicated time points, followed by immediate cooling/fixation in 2%PFS/PBS. The plasma membrane was stained using cell mask deep red stain (1:10.000 in PBS) for 5 min on ice, followed by washing with PBS. Images were acquired using the Inspire software (200.1.388.0) and changes in fluorescence intensity were analyzed using the IDEAS software (6.0.154.0).

### Ceramide detection

Ceramide was analyzed by imaging flow cytometry (Amnis/EMD Millipore). In brief, cells were incubated with ML349 (50 μM, Tocris), GW4869 (20 μM, Sigma-Aldrich) or left untreated for 30 min at RT followed by 20 min cooling down on ice and centrifugation for 4 min 350 x g, 4 °C. 100 ng/mL TNF was incubated for 20 min on ice, followed by 15 min temperature shift to 37 °C. Cells were fixed in 2%PFA/PBS for 15 min on ice, 2x washing and permeabilization in 0.2%Saponin/0.1%BSA/PBS for 15 min on ice. Cells were 2x washed with 0.1%BSA/PBS followed by 30 min incubation with anti-ceramide antibody (clone 15B4, 1:100 in 0.1%BSA/PBS), 2x washing and incubation with anti-mouse-alexafluor488 antibody, diluted 1:200 in 0.1%BSA/PBS for 30 min. Images were acquired using the Inspire software (200.1.388.0) and changes in fluorescence intensity was analyzed using the IDEAS software (6.0.154.0).

### Caspase-3 assay

Cells were incubated with inhibitors for 30 min at 37 °C, followed by 4 h stimulation with 100 ng/ml TNF under cell culture conditions. Cells were then sedimented and lysed (10 mM HEPES [pH 7.4], 5 mM MgCl_2_, 1 mM EGTA, 0.2% NP40, 2 mM AEBSF/Pefabloc, 1 mM DTT (freshly added). 5 μg total cell lysate was then incubated with 100 μl assay buffer (20 mM PIPES [pH 7.2], 100 mM NaCl, 1 mM EDTA, 0.1% CHAPS, 10% Sucrose, 10 mM DTT (freshly added) containing 100 μM zDEVD-AFC (#13420, AAT Bioquest). Increase in fluorescence intensity was monitored at ex: 405 nm/em: 505 nm using an Infinite M200 (Tecan) plate reader at 37 °C.

### Acyl resin assisted capture (acylRAC)

AcylRAC was performed as described by Forrester et al. [23, 24], with minor modifications: 1 × 10^8^ cells per sample were incubated with 100 ng/ml of TNF for 15 min on ice, followed by warming up to 37 °C for the indicated times. Cold PBS was added and cells were sedimented, followed by lysis in 1 ml buffer A (25 mM HEPES [pH 7.4], 25 mM NaCl, 1 mM EDTA, PIC) using sonication (45 s, constant output 2.5, 4 °C) (G. Heinemann, Germany). An aliquot was stored as input in lysis buffer (50 mM TRIS-HCl [pH 7.5], 150 mM NaCl, 1% NP-40, 1% Triton X-100, 1 mM EDTA, 0.25% Na-deoxycholate). Debris was removed by 2x centrifugation (800×g, 5 min, 4 °C) followed by membrane sedimentation for 50 min at 4 °C at 16200×g. The resulting pellet was resuspended in buffer A/0.5% Triton X-100. 1.5 mg protein solution was mixed with the blocking solution (100 mM HEPES [pH 7.5], 1 mM EDTA, 2.5% SDS, 2.5% MMTS (Sigma-Aldrich)) in a 1:2 ratio at 40 °C for 2 h, followed by acetone precipitation. The precipitate was resuspended in 400 μl binding buffer (100 mM HEPES [pH 7.5], 1 mM EDTA, 1% SDS), split equally and added to 0.05 g activated thiopropyl sepharose 6B (GE Healthcare) in binding buffer. One part was treated with hydroxylamine [pH 7.5] the other part with Tris-HCl [pH 7.5], final concentration 0.5 M each. After over night incubation, beads were washed and used for SDS-PAGE.

### Metabolic 17-ODYA labeling

The labeling protocol was adapted from [25]. In brief, to shed TNF-R1 from the cell surface and to trigger transport from the PM cells were washed in PBS and incubated in the presence of 150 μM histamine for 3 h in FCS free medium at cell culture conditions as adapted from Wang et al [26]. Histamine treated and untreated cells were then incubated for 16 h in the presence of 100 μM 17-ODYA (#90270, Cayman), followed by membrane sedimentation as described for acylRAC. The resulting pellet was resuspended in 150 μl 25 mM HEPES [pH 7.4], 25 mM NaCl, 0.5% Triton X-100, PIC. The click reaction was made up fresh with the final concentrations: 500 μM biotin-azide (#13040, Cayman)), 2 mM CuCO_4_, 0.2 mM TBTA (#678937, Sigma Aldrich) and 4 mM ascorbic acid (fresh) in a total volume of 200 μl. After 2 h incubation at RT, proteins were acetone precipitated and then resuspended in 500 μl 50 mM TRIS-HCl [pH 7.5], 150 mM NaCl, 1% NP-40, 1% Triton X-100, 1 mM EDTA, 0.25% Na-deoxycholate. 20 μl Streptavidin-microbeads (#130–048-102, Miltenyi) were added and incubated overnight at 4 °C. After purification via μColums (Miltenyi) and elution using SDS-Sample buffer containing β-mercaptoethanol, 15 μl were used for SDS-PAGE/WB.

### Immunoprecipitation

5 × 10^7^ cells were washed with ice-cold PBS followed by incubation with 100 ng/ml Fc60TNF for 20 min on ice. After the temperature shift for the respective time point, cells were resuspended in 1 ml of IP lysis buffer (50 mM Tris-HCL (pH 7.4), 150 mM NaCl, 1% NP-40, 0.25% Na-deoxycholate, 1% Triton X-100, 1 mM EDTA, Benzonase (Merck) and PIC for 45 min on ice and subsequently 10x sheared using a G21 gauge needle. The lysate was centrifuged 10 min at 10,000 x g and 50 μl protein G Microbeads were added to the supernatant and incubated for 2 h at 4 °C. Upon purification using μ Columns (Miltenyi), 10 μl eluate was analyzed by SDS-PAGE/WB.

### Detection of cleaved Caspase-3 and PARP1

Cells were pre-incubated for 45 min with 50 μM ML349 followed by addition of 100 ng/ml TNF for 6 h and lysis in TNE (1 M Tris pH 8.0, 5 M NaCl, 1% NP40, 0.5 M EDTA; PIC). Proteins were analyzed by SDS-PAGE/WB.

### Analysis of IκB degradation

Cells were cooled on ice for 15 min, followed by incubation with TNF for 15 min, and a temperature shift for the indicated times to 37 °C. Cells were then lysed in 50 mM Tris-HCL (pH 7.4), 150 mM NaCl, 1% NP-40, 0.25% Na-deoxycholate, 1% Triton X-100, 1 mM EDTA, Benzonase (Merck) and PIC, followed by BCA assay and SDS-PAGE/WB.

### SDS-PAGE and Western blot

Where described, protein samples were labeled with lightning red (Serva) diluted 1:50 in protein sample buffer and then separated on anyKD (Biorad) or 12.5% SDS-PAGE gels. Subsequently, total protein staining was analyzed using a Typhoon trio (GE Healthcare). After transfer to PVDF membranes (Carl Roth), membranes were blocked with 5% skimmed milk/TBST, incubated with primary antibodies overnight at 4 °C, followed by washing with TBST and incubation with HRP-conjugated second step antibodies for one hour. Luminescence was detected using ECL reagent and x-ray films (GE Healthcare). Films were scanned and where mentioned, densitometric quantification was performed using ImageJ.

### Expression and activity assay of APT2 and in vitro depalmitoylation

Recombinant APT2 was generated as described [22]. Purified rAPT2 was incubated with crude membrane fractions for 2 h at 37 °C, followed by acylRAC and WB.

### Silencing of APT2

For APT2 silencing, shRNA encoding plasmids (sc-78,672-SH, Santa Cruz) were nucleofected using Amaxa (Lonza), followed by puromycin treatment to generate stable pools.

## Results

### Inhibition of palmitoylation interferes with TNF-R1 internalization

**Fig. 1:**
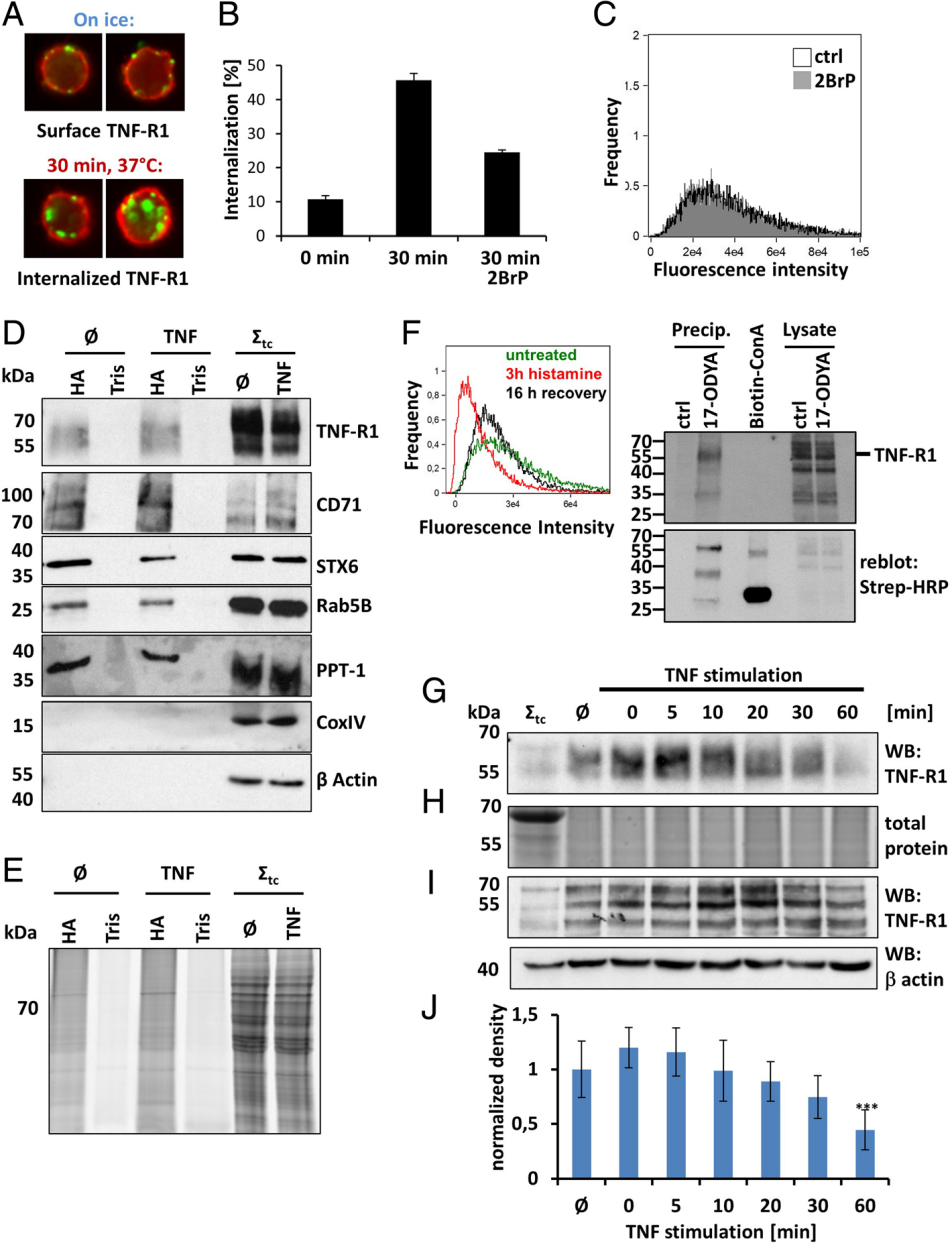
Palmitoylation is involved in TNF signaling. **a** TNF-R1 internalization in response to activation by TNF, quantified by imaging flow cytometry. Representative images of cells kept on ice (upper panel) versus 30 min internalization at 37 °C (lower panel) are shown (TNF/TNF-R1: green, plasma membrane: red). **b** Treatment of cells with the palmitoylation inhibitor 2-bromopalmitate (2BrP; 50 μM) decreases TNF-R1 internalization. Quantification was performed by imaging flow cytometry. Data of three independent experiments +/− SD are shown. **c** The TNF-R1 surface expression at steady state is not reduced in 2BrP treated cells. Quantification was performed by imaging flow cytometry. **d** WB of acylRAC enriched proteins were probed for TNF-R1. Compared to the the total amount in the input, TNF-R1 appears partially palmitoylated as revealed in the captured acylRAC fraction. Known palmitoylated proteins served as positive control: CD71, STX6, Rab5 and PPT1 are present in the input (Σ_tc_) and the (hydroxyl amine) (HA) fractions while Tris lanes show no signals. No difference for untreated (Ø) or 10 min TNF treated cells is apparent. CoxIV and β Actin serve as negative controls. **e** Total protein staining (lightning red) prior to WB. The hydroxylamine (HA) samples contain enriched protein, while the Tris lanes contain negligible protein. Samples were from untreated (Ø) or 10 min TNF treated cells. Σ_tc_ represents the input (cell lysate) for acylRAC. **f** Left panel: TNF-R1 was shed from the cell surface by histamine treatment (red). After 16 h recovery (black curve) the expression resembled the untreated status (green curve). TNF-R1 was labeled using biotinTNF:Streptavidin alexafluor488. Fluorescence intensity mas measured by imaging flow cytometry. Right panel: During recovery phase, 17-ODYA was added to the cells and incorporated into protein palmitoylated within this time frame. After biotinylation of 17-ODYA by click-chemistry and precipitation using streptavidin microbeads, the material was analyzed by WB and compared to lysate as input control. Upper panel: Probing for TNF-R1 showed TNF-R1 in the 17-ODYA treated and the lysate fraction (input). Lower panel: Biotinylated Concanavalin A was used as positive control for biotinylated proteins. **g** TNF-R1 WB from acylRAC samples isolated from 0 to 60 min shows a constant decrease in palmitoylated TNF-R1 up to 60 min. **h** Total protein staining for equal loading prior to WB. **i** WB of total cell lysate corresponding to the fractions of Fig. 1g and h. The total amount of TNF-R1 was constant over time with a slight decrease at 60 min. β Actin serves as loading control. **j** Quantitative WB analysis showing TNF-R1 depalmitoylation kinetics (*n* = 8). All values were normalized to the total TNF-R1 abundance in cell lysates. ***: significant TNF-R1 depalmitoylation (*p* ≤ 0.001)

Internalization and trafficking of TNF-R1 is a prerequisite for the diversification of TNF signal transduction, implicating a putative role of palmitoylation to selectively recruit proteins to distinct signaling platforms in these events [4–7, 27]. In this study, we focused on the monocytic cell line U937, as it responds to to TNF binding with both, NF-κB activation and apoptosis induction without further need for cell death sensitization by i.e. cycloheximide. Application of the commonly used palmitoylation inhibitor 2-bromopalmitate (2BrP) decreased TNF-R1 internalization after 30 min in 50% of the cells (Fig. 1a, b). To analyze this, cells were cooled down on ice in the presence/absence of 2BrP followed by labeling the receptor with biotinylated-TNF/Streptavidin-Alexafluor488. A temperature shift to 37 °C subsequently allowed internalization of the TNF recepors, which was quantified by imaging flow cytometry. Importantly, the surface expression of TNF-R1 at steady state, prior to internalization was not affected by the inhibitor (Fig. 1a, c). These results indicate that S-palmitoylation plays a functional role in TNF signaling.

### Analysis of TNF-R1 palmitoylation

Based on the observation that perturbation of S-palmitoylation by 2BrP altered TNF signaling, we hypothesized that TNF-R1 may be palmitoylated itself, as palmitoylation of other death receptors (i.e. CD95, DR4 and DR6) and implications on their downstream signaling has been reported before [28–30]. To confirm this assumption, we used acylRAC to enrich palmitoylated proteins from untreated and 10 min TNF stimulated cells [24]. Figure 1d and e shows the assessment of the purity of the samples by WB and SDS-PAGE. Probing WB for TNF-R1, we detected constitutive palmitoylation of TNF-R1 while no difference between untreated and TNF treated samples was apparent (Fig. 1d, first panel). Interestingly, by comparing the input fractions (Σ_TC_) and the HA fractions, not all cellular TNF-R1 appears to be palmitoylated. This could be explained by either inefficient capturing during acylRAC or by the assumption that only a certain pool of TNF-R1 is palmitoylated (i.e. receptors at the plasma membrane). As controls, we analyzed the samples for known palmitoylated proteins (CD71, STX6, Rab5B and PPT1) and β Actin and CoxIV as a non palmitoylated controls. Figure 1e depicts the total protein stain to ensure equal loading [31]. The total membrane input (Σ_tc_) +/− TNF contains a variety of proteins, while fewer bands are visible in the hydroxylamine (HA) fraction. The control (Tris) lanes are absent of protein, indicating the enrichment was successful.

To investigate whether endogenous TNF may affect TNF-R1 palmitoylation, we incubated U937 in the presence of an anti-TNF-Fab’ for 14 days, followed by acylRAC/WB analysis. In both, untreated and TNF-Fab’ treated cells, TNF-R1 was found to be palmitoylated (Additional file 1: Figure S1).

Figure 1f shows validation of TNF-R1 palmitoylation by metabolic labeling using the 17-ODYA [25]. TNF-R1 was initially depleted from the PM using histamine treatment [26] (left panel). During recovery, 17-ODYA was incorporated, biotinylated by click chemistry and precipitated, followed by western blot analysis (right panels). We observed no metabolic labeling of TNF-R1 in cells without previous histamine treatment (data not shown). We conclude that TNF-R1 palmitoylation on at least one site occurs during ER/Golgi to PM translocation.

As the signal relayed by TNF-R1 can change over time from proliferation to cell death, we hypothesized that TNF-R1 palmitoylation might also change over time. Indeed, using acylRAC and western blot analysis, we observed that the constitutive TNF-R1 palmitoylation was followed by de-palmitoylation at later time points (Fig. 1g-j). As an attempt to quantify TNF-induced changes in palmitoylation of TNF-R1 and other proteins, we applied similar acylRAC samples to mass spectrometric analysis. The overall changes were modest and could not be quantified (Additional file 4: Table S1). TNF-R1 was also not among the identified proteins.

### Analysis of TNF-R1 palmitoylation sites

**Fig. 2:**
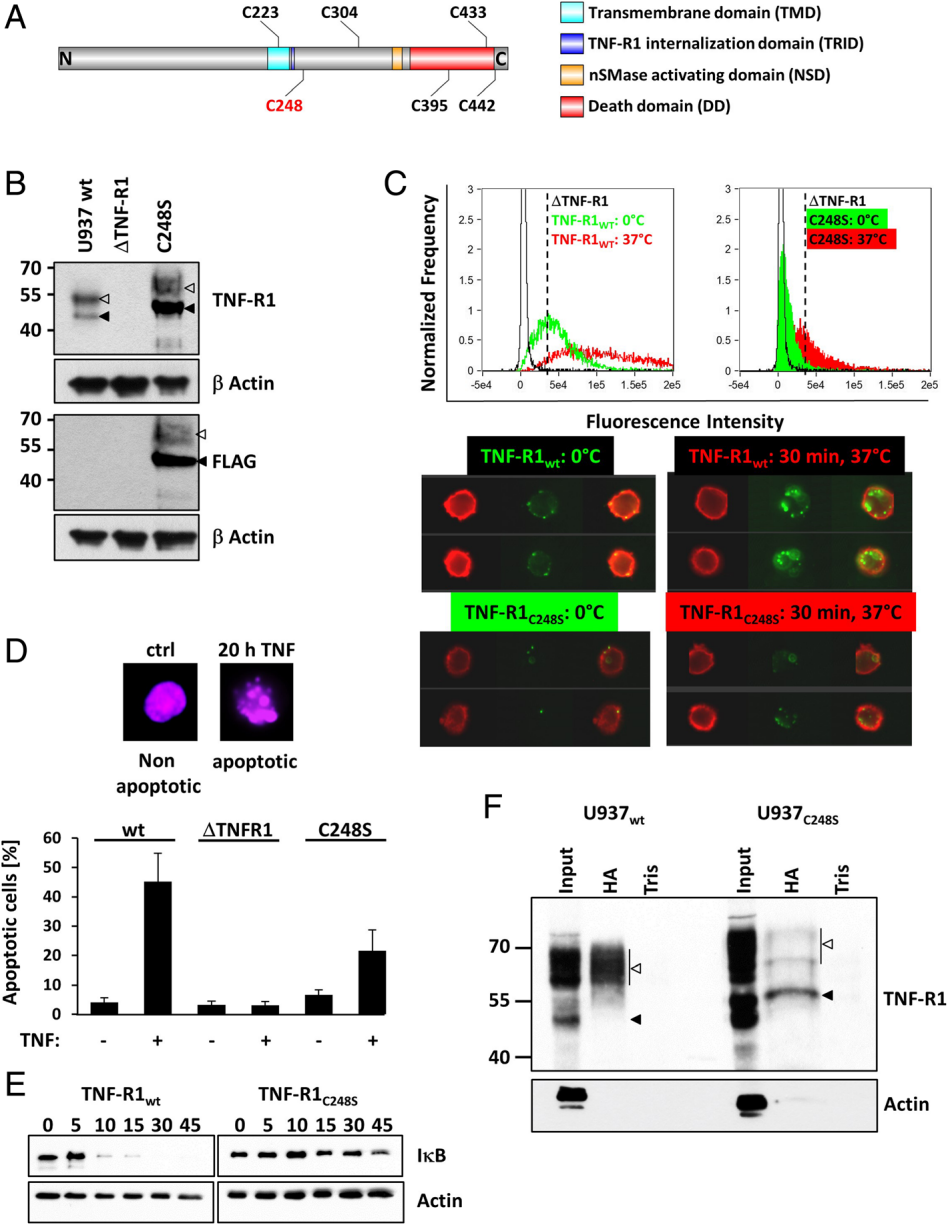
Analysis of the putative palmitoylation site C248. **a** Topology model of TNF-R1 depicting all intracellular cysteine residues and functional domains. The mutated C248 is indicated in red. **b** TNF-R1_C248S_ was expressed in TNF-R1 deficient (ΔTNF-R1) cells. WB analysis of total TNF-R1 expression. The upper panel shows TNF-R1. The third panel shows the FLAG-tag of the construct. Low MW TNF-R1 is indicated by filled arrowheads, high MW TNF-R1 is indicated by empty arrowheads. β Actin serves as loading control. **c** Analysis of TNF-R1 surface expression and internalization. Left histogram: wildtype TNF-R1 expressing cells, right histogram: C248S-TNF-R1 expressing cells. ΔTNF-R1 (black curve), 0 °C/surface TNF-R1 (green) or 30 min, 37 °C/internalized TNF-R1 (red). The shift of the curve/increase in fluorescence intensity is due to TNF-R1 internalization and accumulation in endosomes (TNF-receptosomes). The dashed line marks the medium wt surface expression in both histograms. One representative experiment is shown. Sample images for wt (upper panels) and C248S cells (lower panels) are shown below. The left panels show surface TNF-R1, the right panels show internalized TNF-R1. **d** Apoptosis induction in U937 (wt, ΔTNF-R1 and C248S) analyzed by quantification of nuclear DNA fragmentation. Representative untreated cell with intact nucleus (violet) (upper left panel) and 20 h TNF treated cell with fragmented nucleus (upper right panel). The diagram shows mean values of 3 experiments with up to 10,000 imaged cells. **e** Degradation of IκB was analyzed upon stimulation of wt and TNF-R1_C248S_ expressing cells with TNF by WB. Actin serves as loading control. One representative experiment is shown. **f** AcylRAC from TNF-R1_wt_ (left side) and TNF-R1_C248S_ (right side) expressing cells. Input represents the total membrane fraction used for acylRAC. The WB was probed for TNF-R1 or actin as negative control. Low MW TNF-R1 is indicated by filled arrowheads, high MW TNF-R1 is indicated by empty arrowheads

Comparing the TNF-R1 sequence across species revealed four conserved (C223, 248, 395, 433) and two less conserved cysteine residues (C304 and 442) (Additional file 2: Figure S2). Figure 2a shows a topology model of TNF-R1 including the cysteine residues in its intracellular domain. The occurrence of palmitoylation sites in close proximity to transmembrane domains (TMD) or even within TMD is common between other receptor systems [32]. Palmitoylation of CD95, DR4 and DR6 occurs on cysteines close to the respective TMD [28, 30, 33]. Using the CSS-palm algorithm, Cys248 was predicted as one putative palmitoylation site [34]. Thus, we next generated TNF-R1 knock-out U937 cells (ΔTNF-R1) and re-transfected them with FLAG-tagged C248S mutated TNF-R1 (C248S). TNF-R1 expression was analyzed by WB, showing that C248S cellular expression was higher compared to TNF-R1 in wt U937 cells (Fig. 2b). The two bands recognized by TNF-R1 antibodies are indicated by arrowheads filled (lower MW) empty (higher MW). Conversely, TNF-R1 surface labeling revealed a reduced surface expression of C248S (filled green) compared to TNF-R1_wt_ (green) (Fig. 2c), suggesting that Cys248 is involved in the transport of TNF-R1 to the cell surface. Incubation of labeled cells for 30 min at 37 °C increased fluorescence intensity due to clustering and internalization of the receptors (TNF-R1_wt_: red; TNF-R1_C248S_: filled red Fig. 2c). We next analyzed TNF induced apoptosis, showing that TNF-R1_C248S_ expression restores apoptosis induction compared to ΔTNF-R1 cells, while the percentage of apoptotic cells was less compared to wt cells (Fig. 2d). TNF induced NF-κB activation was quantified by measuring IκB degradation by WB (Fig. 2e), showing that NF-κB activation was reduced.

To analyze whether the cysteine 248 is the only putative palmitoylation site, we performed acylRAC from wt and mutant TNF-R1 expressing cells (Fig. 2f). While TNF-R1 was precipitated in both cell lines, the signal was reduced in TNF-R1_C248S_ expressing cells compared to wt cells. Interestingly, the resulting protein band pattern was altered. While the HA fraction from wt cells showed mainly the high MW variant of TNF-R1 (empty arrowhead), the lower MW variant (filled arrowhead) is much more prominent in TNF-R1_C248S_ expressing cells. Thus, we conclude that C248 is not the only TNF-R1 palmitoylation site but it is required for proper transport of the receptor to the plasma membrane and also for the activation of NF-κB.

Despite large efforts, expression of wildtype or other cysteine mutated TNF-R1 in ΔTNF-R1 U937 cells was toxic and thus, could not be included in the analysis.

### Identification of APT2 as a TNF-R1 palmitoyl thioesterase

**Fig. 3:**
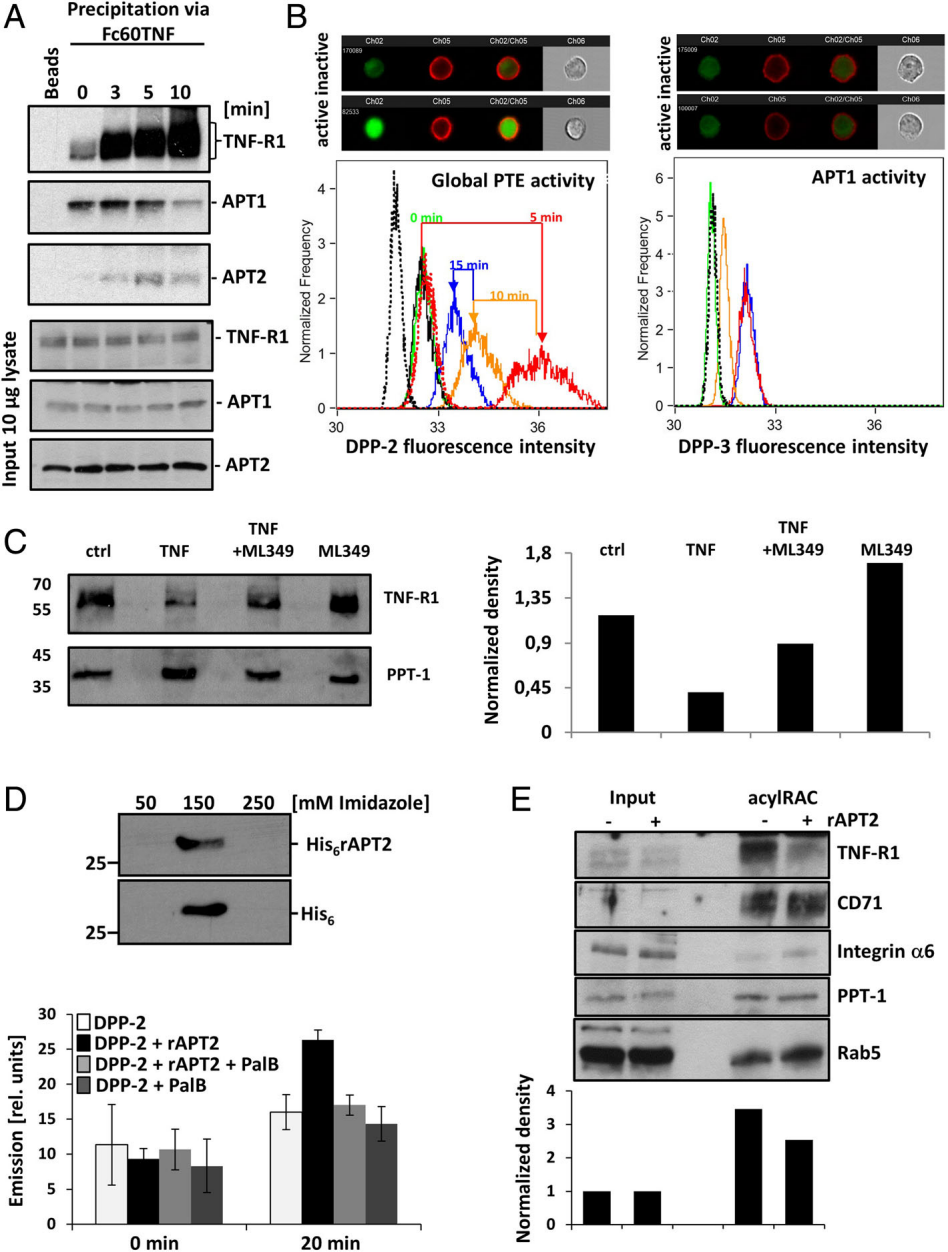
Activation and function of PTE in response to TNF. **a** APT1 and APT2 are part of the TNF-RSC: TNF-R1 was activated, lysed in the presence of detergents and precipitated using Fc60TNF, activating only TNF-R1. Panel one: TNF-R1 displaying the reported increase in K63-ubiquitination in response to TNF [5]. Panel two: Constitutive TNF-RSC:APT1 interaction, decreasing at 10 min. Panel three: Transient recruitment of APT2 to the TNF-RSC. Panels 4–6 show WB with 10 μg of input lysate. **b** Activation of endogenous S-depalmitoylases in response to TNF, analyzed by imaging flow cytometry using fluorescent probes: DPP-2 for global S-depalmitoylation and DPP-3 for APT1 activity. Left panel one: Representative images of inactive APT2. Left panel two: Shows cells with activated fluorescent probe (DPP-2; green) in response to TNF. PM is stained in red. Panel three: histograms representing enzyme activity. Dashed black: untreated cells. Green: addition of DPP-2 and TNF to cells kept on ice. Addition of DPP-2/TNF at 37 °C for 5 min (red), 10 min (orange), 15 min (blue). Black: DPP-2/Palmostatin B (pan PTE inhibitor). Dashed red: DPP-2/ML349 (APT2 selective inhibitor). Right panels: DPP-3 is not activated in response to TNF (same order and color code as A). **c** Left panel: representative WB from acylRAC probed for TNF-R1 at the conditions: no TNF (ctrl), 30 min TNF, ML349 with 30 min TNF, and ML349. PPT-1 serves as loading control. Right panel: WB quantification by densitometry. WB loading control, see Additional file 3: Figure S3. **d** His_6_rAPT2 was expressed in and affinity purified from *E. coli*. WB was probed using APT2 and His antibodies. rAPT2 activity after 20 min incubation at the conditions: DPP-2 (light grey), DPP-2 + APT2 (black), DPP-2 + APT2 + Palmostatin B (medium grey), DPP-2 + Palmostatin B (dark grey). **e** WB of input and acylRAC +/− rAPT2 (30 μM, 1 h 37 °C): Palmitoylation of TNF-R1 decreases while CD71, integrin α6, PPT-1 and Rab5 are not affected. The lower panel shows quantification of the WB for TNF-R1 normalized to PPT-1 levels

As we observed depalmitoylation of TNF-R1, we strived to identify the responsible enzyme. PPT1 has been reported to be localized in lysosomes, while APT1 and APT2 are localized in the cytoplasm [20, 21]. Thus, we focused on APT1 and 2 and performed TNF-R1 signaling complex (TNF-RSC) co-IP using Fc60TNF as bait: Fig. 3a shows an initial constitutive and after 10 min a decreasing interaction of APT1, while APT2 is transiently recruited with a maximum at 5 min. TNF-R1 gets ubiquitinated and thus its molecular weight increases in the same period, as reported before [5, 35]. To investigate the role of APT1 and 2, we treated cells with TNF and measured the enzyme activity in live cells using fluorescent “depalmitoylation probes” (DPPs) and imaging flow cytometry [36, 37]. DPP-2 reports on global depalmitoylase activities, while DPP-3 has increased preference for APT1 [22]. Figure 3b, left side shows the transient TNF/time dependent increase in the fluorescent signal from DPP-2. The peak correlated with the observed transient APT2:TNF-RSC interaction (Fig. 3a). However, DPP-3 showed no change in fluorescent signal upon stimulation, indicating that APT1 is not activated within the same time frame (Fig. 3b, right side). If APT1 also has a role in TNF-R1 signaling, as the co-IP suggests, has to be analyzed.

We next investigated the impact of APT2 inhibition on TNF-R1 palmitoylation using the selective inhibitor ML349 (Fig. 3c) [38]. The WB (left panel) and its quantification (right panel) show TNF-R1 depalmitoylation upon 30 min TNF stimulation. Incubation with ML349 followed by TNF treatment blocked TNF-R1 depalmitoylation, and incubation with ML349 alone led to accumulation of palmitoylated TNF-R1.

We next overexpressed and purified recombinant APT2 (rAPT2) for further functional analysis (Fig. 3d). The upper panel shows enrichment of rAPT2, the lower panel shows analysis of the enzyme activity using the fluorescent probe (DPP-2). To check if APT2 de-palmitoylates TNF-R1 in vitro, incubation with rAPT was performed prior to acylRAC and WB analysis **(**Fig. 3e): Palmitoylation of TNF-R1 decreased while other palmitoylated proteins (CD71, Integrin α6, PPT-1 and Rab5) were not affected. How this selectivity is achieved remains to be clarified.

**Fig. 4:**
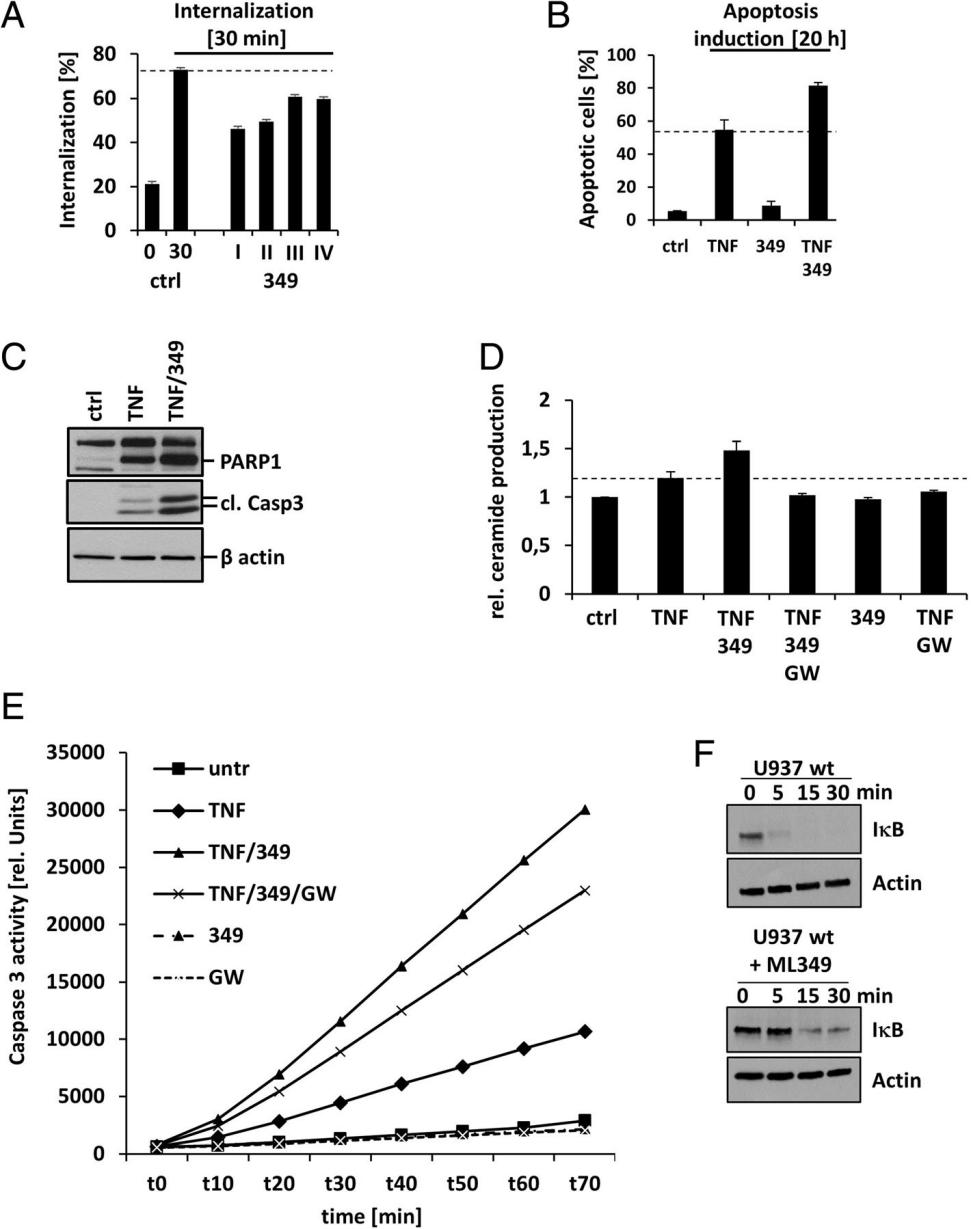
The biological function of pharmacological APT2 inhibition in TNF signaling. The dashed black lines indicate wt apoptosis and internalization levels upon stimulation with TNF. Abbreviation: ML349 (349). All experiments show mean values (+/− SD) of 3–5 independent experiments. **a** TNF-R1 internalization in response to activation by TNF. Ctrl: TNF-R1 internalization increases upon 30 min incubation at 37 °C in the presence of TNF. Incubation with ML349 (I: 50 μM, II: 25 μM, III: 12.5 μM, IV: 6.35 μM) reduced internalization. **b** Apoptosis induction analyzed by quantification of nuclear DNA fragmentation. 20 h TNF treatment increased apoptotic cells. Incubation with ML349 (25 μM) increased apoptosis rate. Higher concentrations of ML349 resulted in excess apoptosis without TNF. **c** Apoptosis induction analyzed by WB. Panel one: PARP1 cleavage. Panel two: active caspase-3. Panel three: actin as loading control. **d** Ceramide production after 10 min stimulation with TNF. TNF induces ceramide formation, which is increased upon APT2 inhibition by ML349. nSMase inhibition by GW4869 (10 μM) protects from ceramide production. **e** Caspase-3 activity was monitored up to 70 min upon addition of 100 ng/ml TNF, 25 μM ML349, 2 μM GW4869. One representative experiment of three measurements in duplicates is shown. **f** Degradation of IκB was analyzed upon stimulation with TNF by WB. Actin serves loading control. One representative experiment is shown

To investigate the in vivo role of APT2 in TNF signaling, cells were then incubated with different concentrations of the inhibitor ML349. First, we observed slightly reduced TNF-R1 internalization from ~ 70% in untreated to ~ 45–60% of the ML349 treated cells (Fig. 4a). Second, analysis of apoptosis unexpectedly revealed enhanced apoptosis for ML349 (Fig. 4b). Probing WB for cleaved PARP1, and cleaved caspase-3 further validated these findings: Both proteins displayed enhanced cleavage upon 6 h co-incubation with TNF and ML349 (Fig. 4c).

Based on previous observations that apoptosis induction requires TNF-R1 internalization, we wondered, how this effect can be explained while TNF-R1 is inhibited using 50 μM ML349. Earlier reports by us and others showed activation of PM/caveolae resident nSMase in response to TNF, resulting in ceramide formation and apoptosis induction [39–43]. In our experiments, TNF also induced increased ceramide levels, which was enhanced by co-incubation with ML349 (Fig. 4d). Inhibition of nSMase using GW4869 blocked ceramide production. Since overnight incubation with GW4869 was toxic, we monitored caspase activity after 4 h TNF stimulation (Fig. 4e), showing that pretreatment with the nSMase inhibitor GW4869 decreased TNF/ML349 stimulated caspase activity.

Pharmacological inhibition of APT2 resulted in reduced NF-κB activation in response to TNF, compared to TNF treated cells in the absence of the APT2 inhibitor (Fig. 4f).

**Fig. 5:**
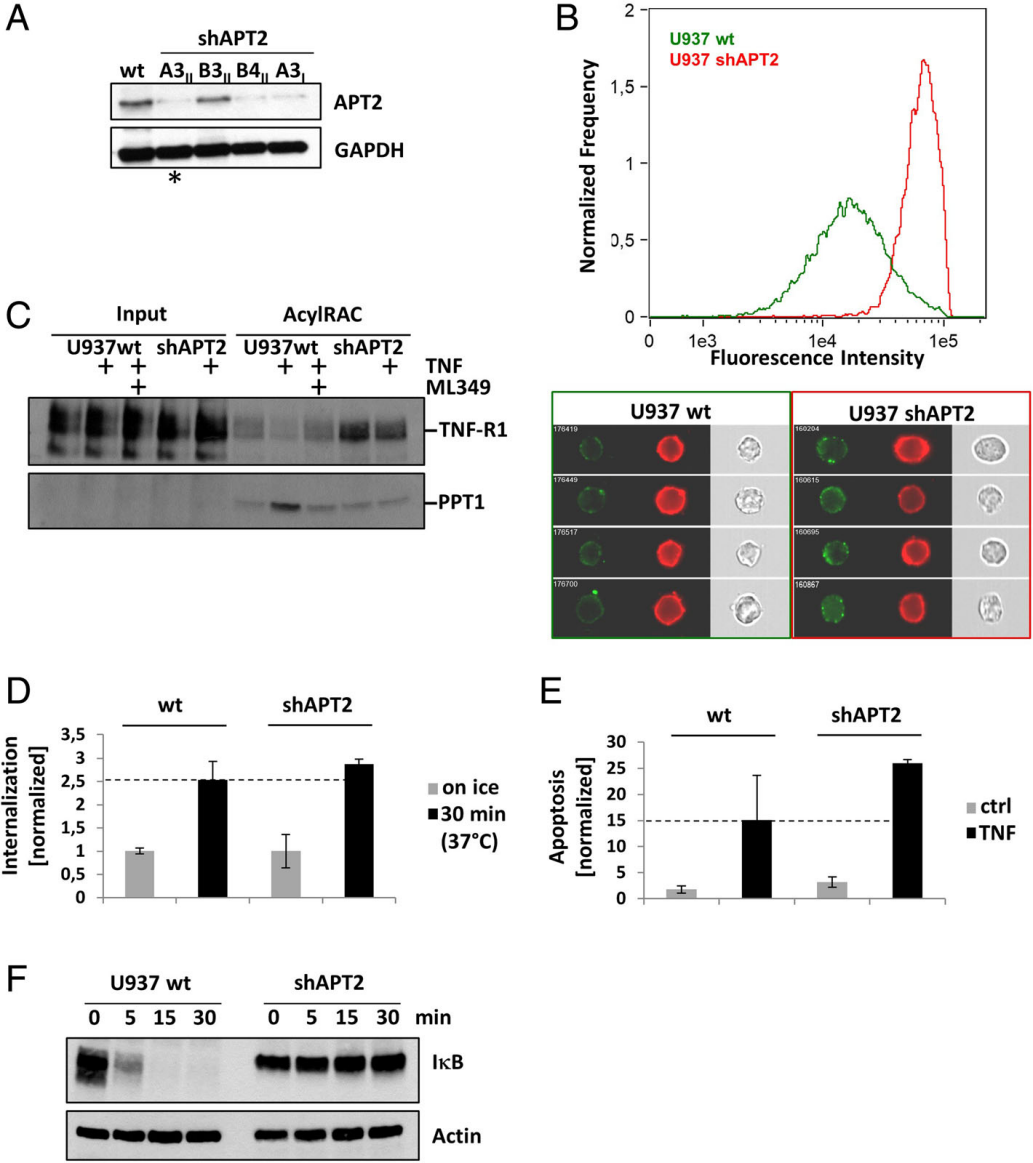
The biological function of APT2 knock-down in TNF signaling. **a** APT2 expression was knocked-down by shRNA and validated by WB. shAPT2 pool A3II (marked by an asterisk) was used for further experiments. GAPDH was used as loading control. **b** The histogram shows that TNF-R1 surface expression is higher (red curve) in shAPT2 cells compared to wt (green curve) cells. Sample images from wt (green box) and shAPT2 (red box) cells are shown below. TNF-R1 is labeled using biotinylated-TNF:Streptavidin-alexafluor488 (green), the plasma membrane is labeled with cell mask (red). **c** TNF-R1 palmitoylation was analyzed by acylRAC. U937 wt cells +/− ML349 were compared to shAPT cells. WB was probed for TNF-R1, PPT1 served as loading control. One representative experiment is shown. **d** TNF-R1 internalization was not affected by shAPT2 knock-down. Mean values of three experiments are shown. **e** TNF induced apoptosis is enhanced in shAPT2 cells. Mean values of three experiments are shown. **f** IκB degradation is inhibited in shAPT2 compared to U937 wt cells. One representative experiment is shown

Down modulation of APT2 using shRNA (Fig. 5a) interestingly enhanced TNF-R1 surface expression compared to wt cells (Fig. 5b). The inhibitory effect on TNF-R1 depalmitoylation was similar to inhibition of APT2 using ML349 (Fig. 5c). Down modulation of APT2 did not reduce TNF-R1 internalization (Fig. 5d) and increased the apoptotic response (Fig. 5e) which is in line with the effects of ML349 shown before in Fig. 4b. Interestingly, treatment of U937 cells with TNF resulted in slightly stronger signals for PPT-1 in acylRAC samples, which was also apparent in Fig. 3c.

Activation of the NF-κB pathway was also blocked in shAPT2 cells (Fig. 5f), similar to pharmacological APT2 inhibition.

## Discussion

Several earlier studies by us and others showed that the biological outcome of TNF signaling depends on the subcellular localization of the activated TNF-R1 [8, 15, 16]. Receptors residing in the plasma membrane signal for cell survival via “complex I” formation while endocytosis of TNF-R1 changes signaling capacity to induction of cell death by DISC recruitment / “complex II” formation [2, 4, 44]. These diametrically opposed effects have to be tightly regulated, to ensure controlled biological outcomes. While we recently showed that TNF-R1 has to be K63-ubiquitinated to allow its internalization and thereby, switching to cell death signaling [5] others showed that linear ubiquitination of TNF-R1 is essential for NF-κB signaling [45]. How the formation of the distinct signaling complexes is orchestrated in the correct spatio-temporal context is still unknown.

S-palmitoylation of proteins is a reversible mechanism to modulate protein-protein and protein-membrane interactions, mainly by changing their affinity for membranes/lipid rafts/detergent resistant membranes (DRM) [20, 46, 47]. While many surface proteins have been described to be palmitoylated, this has not been investigated in the context of TNF-R1 signaling. For three other members of the TNF-receptor-superfamily (CD95, DR4 and DR6) palmitoylation has been reported. Intriguingly, palmitoylation of CD95 and DR4 is required for lipid raft localization and cell death signaling [28, 29], palmitoylation of DR6 prevents association with lipid rafts [30].

Three groups reported that TNF-R1:lipid raft association is essential for ERK and NF-κB regulation [35, 48, 49], suggesting a possible involvement of palmitoylation. Other groups reported TNF-R1 association and cell death induction with caveolae-like structures, which represent a subgroup of lipid rafts/DRM [42, 50, 51]. In addition, D’Alessio and colleagues showed that TNF-R1 shedding by TACE is also dependent on lipid raft microdomains [52], which may also regulate the recently described subsequent TNF-R1 intramembrane ripping by γ-secretase [27]. Also, changes in the lipid raft proteome in response to TNF point towards a role of palmitoylation of regulatory elements in this phenomenon [53, 54]. However, the role of lipid rafts for TNF-R1 signaling is not fully understood and may depend on the cell type investigated. In contrast to the HT1080 cell line [35], TNF-R1-induced apoptosis has been reported to depend on lipid rafts in the U937 myeloid cell line [49]. In primary mouse macrophages, lipid rafts/caveolae appear to be important for transducing TNF-R1 signaling to the MAPK/ERK pathway but not to NF-κB activation [50]. A selective lipid raft dependency of TNF-R1 signaling to p42 MAPK/ERK was observed in primary mouse macrophages [49], but in human airway smooth muscle cells NF-κB and MAPK activation by TNF was found to be independent of lipid rafts [55]. In the human endothelial cell line EA.hy926, TNF-R1-mediated activation of phosphatidyl-inositol 3-kinase (PI3K), but not of NF-κB, seems to originate from caveolae after interaction of TNF-R1 with caveolin-1. From these caveolae, TNF-R1 can also be internalized in a clathrin independent manner [56]. By contrast, disruption of lipid rafts in HT1080 fibrosarcoma blocked NF-κB activation and sensitized cells to apoptosis [35]. Ali and colleagues recently showed that TNF mediated necrosome formation occurs in caveolin-1-containing DRM [57]. We recently identified the anti-epilepsy drug Phenhydan® as potent inhibitor of both, TNF-R1 mediated NF-κB and cell death signaling by influencing lipid raft formation [58]. Thus, redistribution of TNF-R1 into DRM/lipid rafts and non-raft regions of the membranes seems to regulate the diversity of signaling responses by TNF in various cell types, but the quality of signals transduced from lipid rafts varies significantly between different cell lines.

We identified constitutive palmitoylation of TNF-R1 but the number, exact sites and which PAT are involved is still unclear and has to be unraveled in further studies. In an acylRAC proteome analysis of TNF-R1 CRISPR/Cas9 edited cells, which is part of a different project, we identified one TNF-R1 peptide (data not shown). Analysis of acylRAC samples by MS in this study did not result in identification of TNF-R1 peptides. The low coverage of peptides identified by mass spectrometry is in line with two earlier reports [59, 60], as well as with our own unpublished proteomic analyses of TNF-receptosomes, suggesting that it is challenging to detect TNF-R1 by MS. As an attempt to directly show palmitoylation of TNF-R1 by MS, TNF-R1 IP was performed followed by MS analysis selectively searching for TNF-R1 peptides with and without putative + 238,22 Da mass shift by palmitoylation. Despite a TNF-R1 sequence coverage of 39%, detection of peptides containing any of the intracellular cysteines was lacking (data not shown).

Mutagenesis of the putative palmitoylation site C248 led to high overall cellular TNF-R1 expression but reduced TNF-R1 surface expression. IκB degradation was blocked in C248S cells. Compared to TNF-R1 knock-out cells cell death was restored but on a lower level. We assume that C248 palmitoylation is required for Golgi to PM transport, which is in line with the report by Wang et al., showing that surface TNF-R1 is shed from the cell surface in response to histamine and subsequently replenished by TNF-R1 from an intracellular Golgi pool [26]. This is supported by our observation that upon PM-depletion of TNF-R1 by histamine, palmitoylation of the receptor was validated by metabolic labeling with 17-ODYA. In 2009, Rossin et al. claimed that DR4 but not TNF-R1 and DR5 are palmitoylated, using metabolic labeling with radioactive palmitate [28]. In this study TNF-R1 was probably not detected due to the unfitting timeframe for metabolic labeling or due to the fact that only a fraction of all cellular TNF-R1 is palmitoylated at a time.

Altered subcellular distribution of TNF-R1 may also result in reduced glycosylation/sialylation of TNF-R1 [6, 61]. This could explain the different band patterns observed by acylRAC/WB obtained from wt and C248S cells. Han and colleagues showed that lacking TNF-R1 N-glycosylation reduced TNF binding to TNF-R1 diminishing downstream signaling [61]. Holdbrooks and colleagues reported that α2–6 sialylation of TNF-R1 inhibits TNF-induced TNF-R1 internalization and apoptosis induction [6]. Lacking sialylation could result in enhanced basal TNF-R1 internalization, which could also explain the reduced surface expression, that we observed in cells carrying a mutation in the C248 palmitoylation site in TNF-R1.

We found that APT2 is involved in TNF-R1 de-palmitoylation which may be prerequisite for lipid raft translocation and NF-κB activation as reported by others [35, 49]. This would be in line with our recent report, showing that interfering with membrane composition using Phenhydan®, also blocks NF-kB activation [58]. Pharmacological APT2 inhibition resulted in reduced internalization while lowering the inhibitor concentration reversed this effect. This might be explained by the effect that other PTE like APT1 are activated to compensate for APT2 function and/or APT2 has further roles in TNF-R1 signaling [62]. Unexpectedly, we observed an increase in apoptosis upon APT2 inhibition and shRNA mediated knock-down. We and others have shown that TNF-R1 activation induces pro-apoptotic ceramide production by plasma membrane resident nSMase [39–41]. Decreased TNF-R1 internalization by APT2 inhibition led to enhanced ceramide levels, which could be blocked by nSMase inhibition. Palmitoylation of nSMase-2 has been described before [63]. Moylan and colleagues showed that nSMase-3 can be activated by TNF in detergent resistant membranes, leading to ROS and ceramide production [43]. TNF-R1 is linked to nSMase by EED, which in turn interacts with integrins [64]. Palmitoylation of Integrinα6 by zDHHC3 has been described before [65]. In glioma cells, Integrinα6β1 prevents TNF induced apoptosis [66]. Palmitoylation of TNF-R1, nSMase and integrins might allow pre-assembly of these proteins in the same membrane compartment.

We also observed APT1:TNF-R1 interaction by co-IP, while the function of APT1 in the TNF-RSC remains to be investigated. The lysosomal PTE PPT1 has recently been linked to TNF signaling by showing that PPT1/Cln-1 deficiency results in resistance to TNF induced apoptosis induction which is in line with our observations that TNF-receptosomes have to maturate into multivesicular bodies and lysosomes for full apoptosis induction [67]. Interestingly, we observed higher PPT1 levels in acylRAC samples upon TNF stimulation. Direct PPT1:TNF-R1 interaction has not been observed.

Earlier reports showed that transmembrane TNF is palmitoylated which regulates its affinity to TNF-R1 [68, 69]. FasL and also the putative DR6 ligand APP require palmitoylation for their biological function [70–72]. We ruled out a possible role for endogenous TNF on TNF-R1 palmitoylation by co-culture with TNF targeting Fab.

## Conclusion

**Fig. 6:**
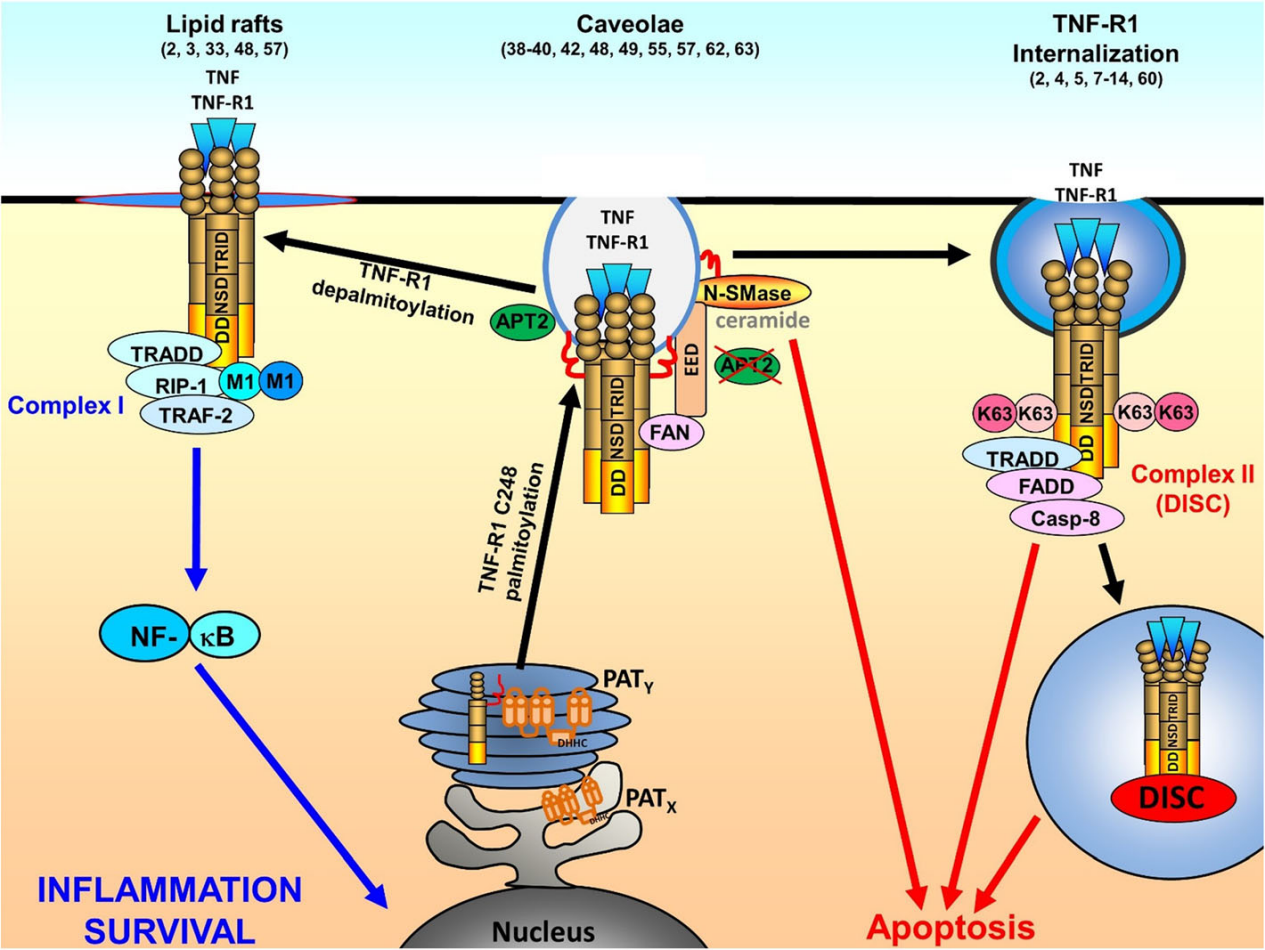
Model. TNF-R1 is palmitoylated in the ER (PAT_x_) or Golgi (PAT_y_) and transported to caveolae in the plasma membrane. After TNF binding, TNF-R1 is either de-palmitoylated by APT2, resulting in translocation to another lipid raft compartment, complex I formation and signaling for NF-κB activation. Alternatively, TNF-R1 may translocate out of caveolae and TNF-receptosomes are formed by clathrin dependent internalization. This allows complex II/DISC formation by intracellular maturation. In case of APT2 depletion/inhibition, activated TNF-R1 induces nSMase dependent ceramide production in caveolae which triggers cell death

Based on our results and reports from other groups, we propose the following model of de-palmitoylation events in the regulation of TNF-R1 signal transduction (Fig. 6): TNF-R1 is palmitoylated in the Golgi to allow transport to distinct plasma membrane domains (i.e. caveolae). Activation of TNF-R1 requires TNF-R1 de-palmitoylation by APT2, allowing translocation to another lipid raft compartment and recruitment of the “complex I” proteins TRADD, RIP-1 and TRAF2, which is also palmitoylated in response to TNF. This induces NF-κB activation and cell survival. Capturing activated TNF-R1 in its steady state plasma membrane/caveolae localization after APT2 inhibition results in a strongly enhanced activation of nSMase and ceramide production, resulting in a shift to apoptosis. Internalization of TNF-R1 which may occur from both caveolae and unstructured plasma membrane is neither affected by C248S mutation nor APT2 down modulation.

In sum, our study demonstrates that palmitoylation represents a novel layer of regulation in TNF-R1 signaling. Observation of TNF-R1 ubiquitination [5, 35, 45, 73, 74], glycosylation [6, 61] and TNF-R1 phosphorylation [75, 76], highlight the importance of posttranslational modifications for proper TNF signaling to maintain homeostasis. Further in-depth characterization and understanding their role in TNF but also in TRAIL and FasL signal transduction may provide a means to interfere and modulate signaling on a new level and might provide access for pharmaceutical intervention for future disease treatment (i.e. chronic inflammatory diseases and cancer).

## Additional files

Additional file 1: **Figure S1.** Endogenous TNF does not affect TNF-R1 palmitoylation. U937 cells were cultured for 14 days in the presence of 0.5 μg/ml anti-TNF-Fab’-Fragment or left untreated (ctrl). Prior to stimulation with exogenous TNF (10 min, 100 ng/ml), cells were washed and acylRAC/WB was performed. One representative experiment is shown. (JPG 201 kb)

Additional file 2: **Figure S2.** TNF-R1 sequence alignment. Alignment of TNF-R1 amino acid sequence from different species (part of the N-terminus is not shown). Red box: conserved Cys residues representing possible palmitoylation sites. Blue box: TMD. (JPG 812 kb)

Additional file 3: **Figure S3.** Role of APT2 in TNF signaling. Loading control for Fig. 3c. Equal amounts (30 μg) of the input material used for acylRAC are shown and WB was blotted for TNF-R1. (JPG 171 kb)

Additional file 4: **Table S1.** Proteins identified by mass spectrometry after enrichment of palmitoylated proteins by acylRAC after 0–60 min TNF incubation. The sample processing and data analysis is described in table. (XLSX 346 kb)

## Data Availability

All data generated or analyzed during this study are included in this published article.

## Abbreviations

17-ODYA: 17-Octadecynoic acid
2BrP: 2-bromopalmitate
acylRAC: Acyl resin assisted capture
APT1/2: Acyl-protein thioesterase 1/2
aSMase: Acid sphingomyelinase
CD95: Cluster of differentiation 95 (Fas ligand receptor)
c-IAP1: Cellular inhibitor of apoptosis 1
CtsD: Cathepsin D
Cys/Cxxx: Cysteine
DD: Death domain
DISC: Death inducing signaling complex
DPP: Depalmitoylation probe
DR (4/5/6): Death receptor (4/5/6)
DRM: Detergent resistant membranes
ER: Endoplasmic reticulum
HA: Hydroxyl amine
IκB: Inhibitor of kappa B
MS: Mass spectrometry
NF-κB: Nuclear factor ‘kappa-light-chain-enhancer’ of activated B-cells
nSMase: Neutral sphingomyelinase
PARP1: Poly (ADP-Ribose)-Polymerase 1
PAT: Palmitoyl acyltransferase
PM: Plasma membrane
PTE: Palmitoyl thioesterase
RIP1: Receptor interacting kinase 1
shRNA: Small hairpin RNA
tBID: Truncated BH3-interacting domain death agonist
TMD: Transmembrane domain
TNF: Tumor necrosis factor
TNF-R1/2: Tumor necrosis factor receptor 1 / 2
TRADD: TNF-R1-associated death domain protein
TRAF2: TNF-receptor associated factor 2
WB: Western blot
wt: Wildtype
ΔTNF-R1: TNF-R1 knock-out
Σ_tc_: Total membrane input
