# Supplementary figures and images for: Palmitoylation is required for TNF-R1 signaling
Source: Cell Commun Signal. 2019 Aug 5;17:90. doi: 10.1186/s12964-019-0405-8 (PMC6683503; doi:10.1186/s12964-019-0405-8)

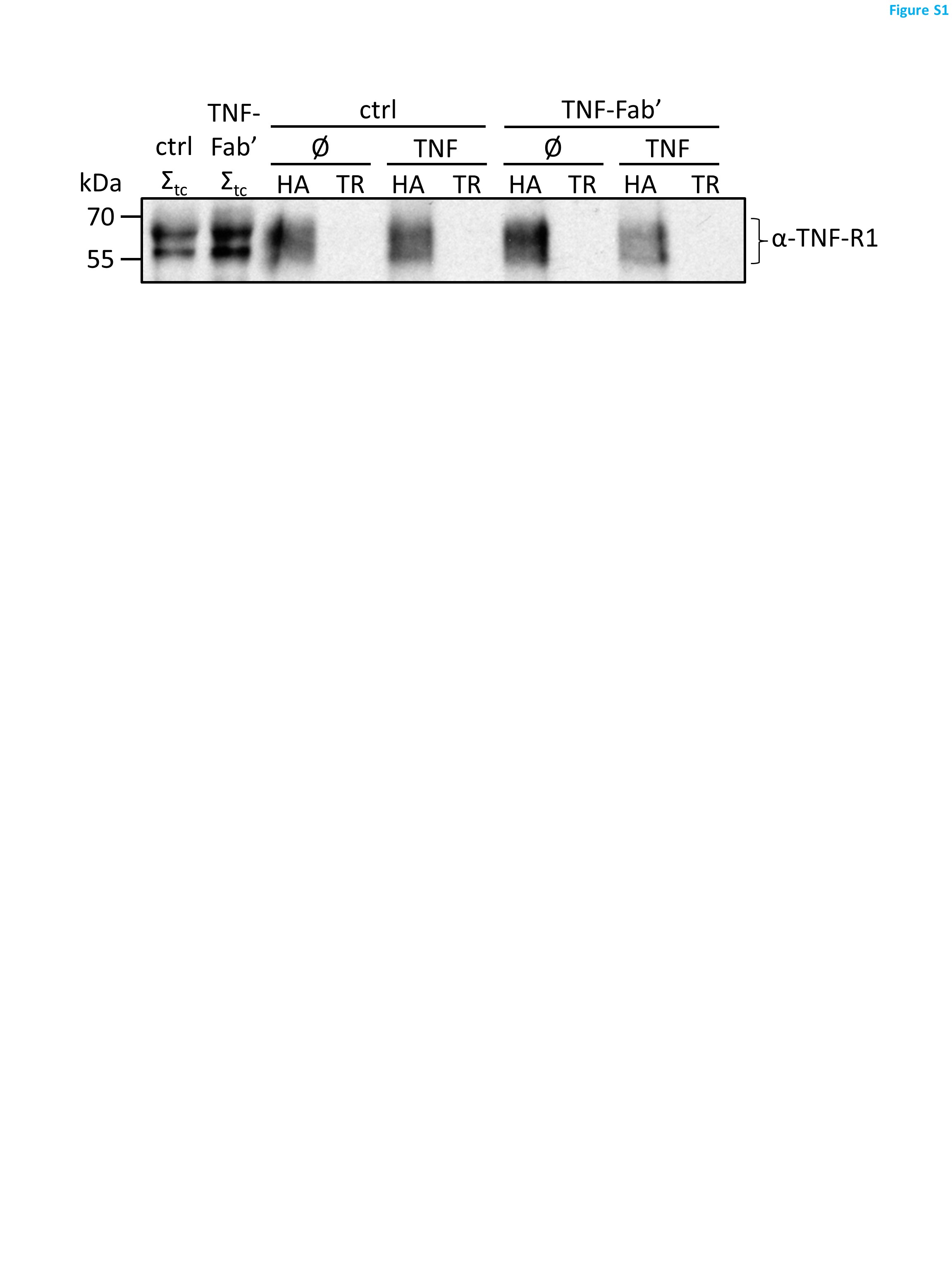

Supplement: Supplementary file 1 — Figure S1. Endogenous TNF does not affect TNF-R1 palmitoylation. U937 cells were cultured for 14 days in the presence of 0.5 μg/ml anti-TNF-Fab’-Fragment or left untreated (ctrl). Prior to stimulation with exogenous TNF (10 min, 100 ng/ml), cells were washed and acylRAC/WB was performed. One representative experiment is shown. (JPG 201 kb) [file 12964_2019_405_MOESM1_ESM.jpg]

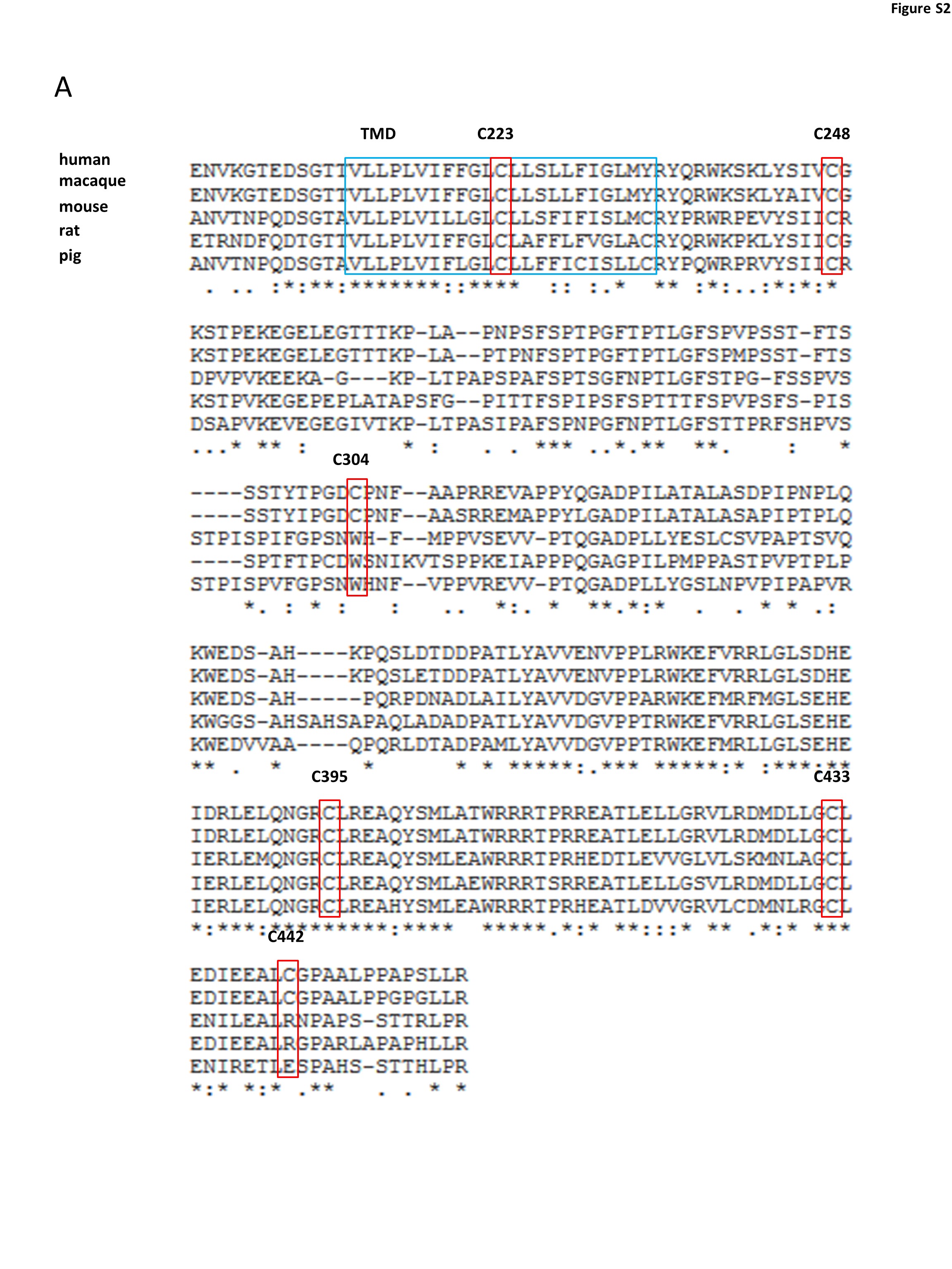

Supplement: Supplementary file 2 — Figure S2. TNF-R1 sequence alignment. Alignment of TNF-R1 amino acid sequence from different species (part of the N-terminus is not shown). Red box: conserved Cys residues representing possible palmitoylation sites. Blue box: TMD. (JPG 812 kb) [file 12964_2019_405_MOESM2_ESM.jpg]

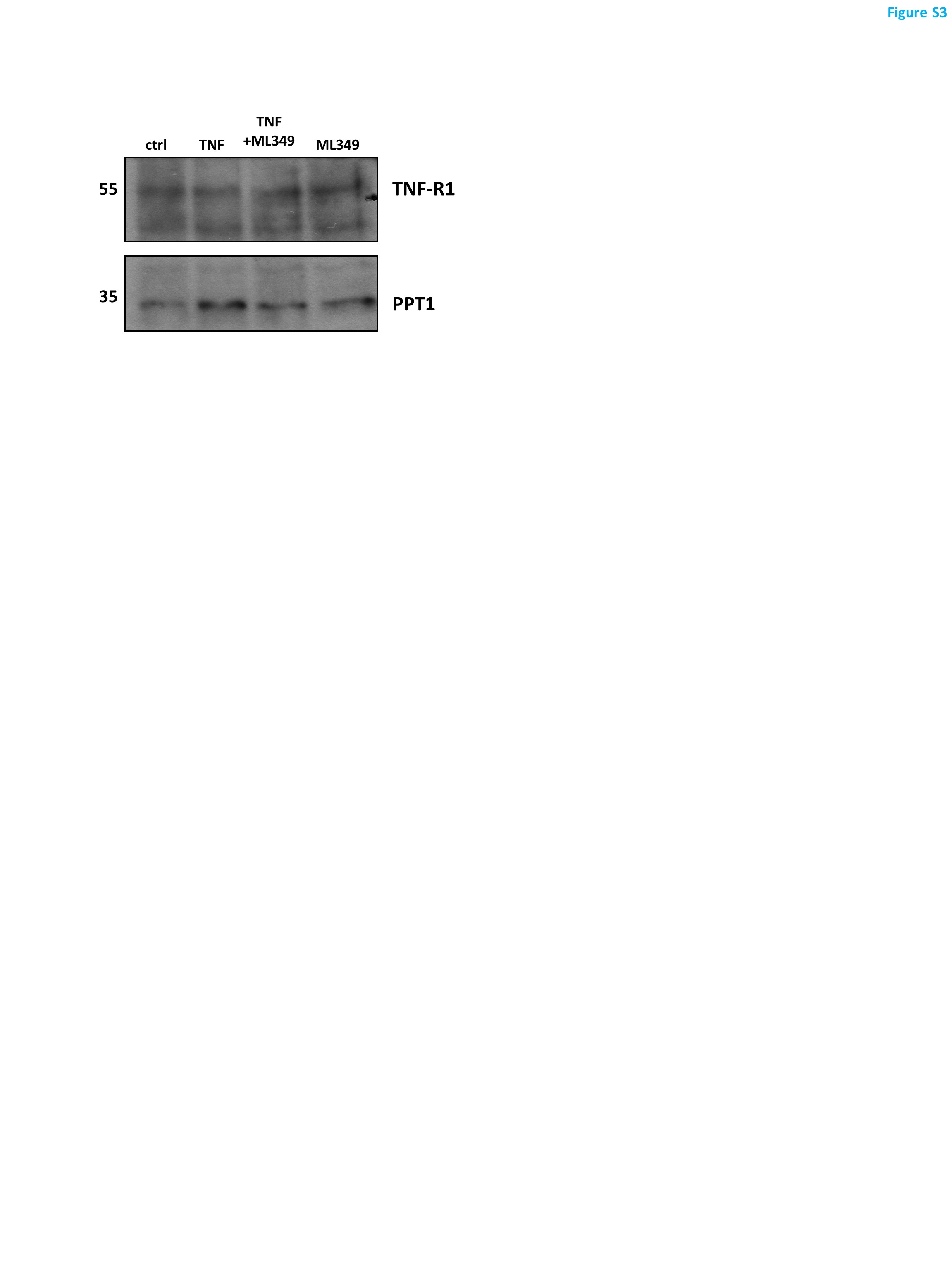

Supplement: Supplementary file 3 — Figure S3. Role of APT2 in TNF signaling. Loading control for Fig. 3c. Equal amounts (30 μg) of the input material used for acylRAC are shown and WB was blotted for TNF-R1. (JPG 171 kb) [file 12964_2019_405_MOESM3_ESM.jpg]
